# The Relationship Between Gambling Event Frequency, Motor Response Inhibition, Arousal, and Dissociative Experience

**DOI:** 10.1007/s10899-020-09955-0

**Published:** 2020-06-14

**Authors:** Andrew Harris, Georgina Gous, Bobbie de Wet, Mark D. Griffiths

**Affiliations:** 1 International Gaming Research Unit, Psychology Department, Nottingham Trent University, 50 Shakespeare Street, Nottingham, NG1 4FQ UK; 2 Psychology Department, University of Lincoln, Lincoln, UK

**Keywords:** Gambling, Event frequency, Arousal, Speed of play

## Abstract

Speed of play has been identified as a key structural characteristic in gambling behaviour, where games involving higher playing speeds enhance the experience of gambling. Of interest in the present study is the consistent finding that games with higher event frequencies are preferred by problem gamblers and are associated with more negative gambling outcomes, such as difficulty quitting the game and increased monetary loss. The present study investigated the impact of gambling speed of play on executive control functioning, focusing on how increased speeds of play impact motor response inhibition, and the potential mediating role arousal and dissociative experience play in this relationship. Fifty regular non-problem gamblers took part in a repeated-measures experiment where they gambled with real money on a simulated slot machine across five speed of play conditions. Response inhibition was measured using an embedded Go/No-Go task, where participants had to withhold motor responses, rather than operating the spin button on the slot machine when a specific colour cue was present. Results indicated that response inhibition performance was significantly worse during faster speeds of play, and that the role of arousal in this relationship was independent of any motor priming affect. The implications of these findings for gambling legislation and gambling harm-minimisation approaches are discussed.

## Introduction

Problem gambling is regularly identified as being associated with rapid speeds of play in games (Harris and Griffiths 2018). It has been established in the literature that higher playing speed is one of the key features which attracts gamblers to games, and as a result, has an increased likelihood of being associated with gambling-related harm, as well as higher levels of general gambling participation (Parke and Griffiths 2007). Faster speeds of play have been found to generate a significantly increased excitement rating when compared to slow speed games, with significantly higher preference ratings for rapid speed machines (Delfabbro et al. 2005).

Existing literature on gambling has determined an inextricable association between speed of play and event frequency, which refers to the number of gambling events within a given time period (Griffiths and Auer 2013). In any given gambling game, event frequency represents the time interval between successive wagers—for example, a weekly lottery has an event frequency of once a week, whereas an EGM (electronic gaming machine) that spins 10 times a minute has an event frequency of 6 s. There is growing evidence which suggests that to problem gamblers, games with fast speed of play (such as EGMs) are especially appealing (Griffiths 2008; Harris and Griffiths 2018). Additionally, it has repeatedly been observed that for problem gamblers who seek treatment or interventions, gambling which involves a faster speed of play (such as EGMs) are identified as a major contributor for their disordered gambling (e.g., Griffiths 2008; Meyer et al. 2009; Turner and Horbay 2004).

There are currently multiple theoretical propositions in existence which attempt to account for the relationship between disordered gambling and participation in high event frequency gambling. Gray’s (1970) Reinforcement Sensitivity Theory (RST) may explain why problem gamblers prefer games with fast speeds of play. RST suggests that a unique system known as the Behavioural Approach System (BAS) creates a behavioural motivation to seek out reward (Gray 1981, 1991). The reward which follows reinforces the behaviour because it is pleasurable and leaves the individual with an increased sensitivity to potential future rewards. It also renders behavioural extinction difficult. Therefore, it is perhaps predictable that gamblers with higher sensitivity to rewards experience increased attraction to games with fast speed of play because such games possess higher event frequency and consequently carry the potential for increased levels of reward across less time.

Developing problem gambling due to reward-punishment processing abnormalities in the brain is a potential risk for individuals who possess dopaminergic-functioning abnormalities in addition to ventro-medial prefrontal cortex structures (Goudriaan et al. 2004). It has been argued by Pickering and Gray (1999) that this reward-punishment system is driven by sensorimotor and prefrontal regions in conjunction with dopaminergic fibres ascending from both the substantia nigra and ventral tegmental areas that innervate the basal ganglia within the brain. It has been reported that when faced with reduced speeds and limited sounds during gameplay, pathological gamblers (compared to nonpathological gamblers) experience significantly decreased ratings of enjoyment, excitement, and tension reduction (Loba et al. 2002). These pathological gamblers additionally reported struggling more to stop gambling when speed of play and accompanying sound was increased.

Gray (1991) argues that one protective factor in the persistence of risk-taking behaviour is sensitivity to loss or punishment. Games with increased event frequencies also deliver increased rates of loss, which theoretically could be a deterrent for gamblers with increased levels of sensitivity to punishment. However, research does not support this claim when gamblers experience high levels of sensitivity to *both* reward and punishment. It has been argued that in order to alleviate the negative emotional mood state caused by loss, gamblers simply engage in further gameplay as result of the increased sensitivity to punishment, resulting in loss-chasing behaviours (Gaher et al. 2015). Consequently, RST predicts that attraction to and persistence on games with increased event frequencies will be higher among individuals with high reward sensitivity and/or punishment sensitivity.

Gambling is an activity in which harm may occur and requires the persistent updating of goals and behavioural adjustments. Therefore, it may be harmful for gambling features, such rapid event frequency, to facilitate dissociative experiences. For example, the organization of gambling stimuli in rapid succession paired with reward in the form of fast, rhythmic, and continuous responses delivered by EGMs facilitates a mental state which is deeply immersive and capable of limiting the intake of peripheral information by lowering conscious awareness. As a result, gamblers may experience a dissociative state which has been found to be pleasurable to the gambler (Griffiths et al. 2006) and enabled by the fast speed of play. These dissociative experiences limit the need for conscious decision-making and provide negative reinforcement to gamble through tension reduction in the form of an escape from wider psychological distress (Fang and Mowen 2009). It has been argued that routine activation of behaviour via the sacrificing of top-down executive control is maladaptive in specific situations, such as those which require decision-making and planning or where danger is a potential risk (Norman and Shallice 1986).

Negative consequences as a result of behavioural perseverance is a sign of disordered gambling (Thompson and Corr 2013) as well as a range of other clinical disorders such as borderline personality disorder (Davey 2008) and psychopathy (Newman et al. 1987). Many types of gambling afford a continuous and rapid pace of play with high event frequencies which could interfere with the gambler’s ability to understand new information, make behavioural adjustments, and update goals in order to avoid negative consequences. If an opportunity is not afforded to a gambler to pause and take stock between gambling events, the likelihood is that they will respond adaptively to punishment decreases (e.g., financial loss). Consequently, less opportunity for reflection is afforded by high event frequency games and as such are increasingly likely to lead to poorly adapted behaviour leading to problem gambling in some individuals. Interestingly, when problem gamblers are obligated to take a five second pause between gambling events, their persistent in gambling is no longer than non-problem gamblers, as shown in experiments by Corr and Thompson (2014) and Thompson and Corr (2013). It should be noted that it is not clear whether this effect is the result of an increase in reflection time or a decrease in the enjoyment of the game generated by the pause (factors which may not be mutually exclusive).

Speed of play has been identified as a key structural characteristic in gambling behaviour (Harris and Griffiths 2018), with games involving higher playing speeds enhancing the experience of gambling (Thompson et al. 2009). Regular gamblers have been found to gamble significantly more per minute than non-regular gamblers (Griffiths 1994). However, evidence which explains why there is an association between fast speed of play and disordered gambling remains largely correlational, despite theoretical models presenting high face validity in explanation of this association. It can be argued that the existing empirical associations assume that an extensive knowledge-base has previously been established, despite this evidence being weak, which could be harmful to scientific research investigating disordered gambling and speeds of play. As such, it is the aim of the present paper to fill the gaps in the present understanding of high event frequency gambling with the goal of facilitating the development of gambling harm-minimisation approaches.

### Research Aims and Hypotheses

The first aim of the present study was to experimentally investigate the impact of gambling speed of play on a gambler’s ability to withhold motor responses during gambling. It was hypothesised that as event frequency increases on electronic slot machine simulators, response inhibition performance would decrease (H1). The second aim was to investigate the psychological factors that predict the relationship between gambling event frequency and response inhibition performance. It was hypothesized that subjective arousal would increase at faster speeds of play (H2), and that increased arousal would be predictive of poorer response inhibition performance (H3). The third aim was to investigate if the inclusion of brief pauses in play between gambling events allow for adaptive response modulation (i.e., allow gamblers to adapt their behaviour to avoid erroneous responses). Existing research demonstrated that the imposition of a simple short delay between gambling events in a computerised card game strengthened inhibitory control processes (see Thompson and Corr 2013). However, the inclusion of brief pauses in play as a means to facilitate inhibitory control processes has yet to be investigated in gambling games with high event frequencies such as slot machine gambling. It is predicted that by providing a short pause following presentation of a gambling result will provide a refractory period that allows executive control systems to exercise control over actions, actions that may otherwise have been automatically and impulsively executed by the provision of a new gambling event. Therefore, it was hypothesised that inclusion of brief pauses in play during slot machine gambling would improve response inhibition performance by facilitating proactive motor control, as demonstrated by an increased reaction time (H4).

## Method

### Design

**Fig. 1:**
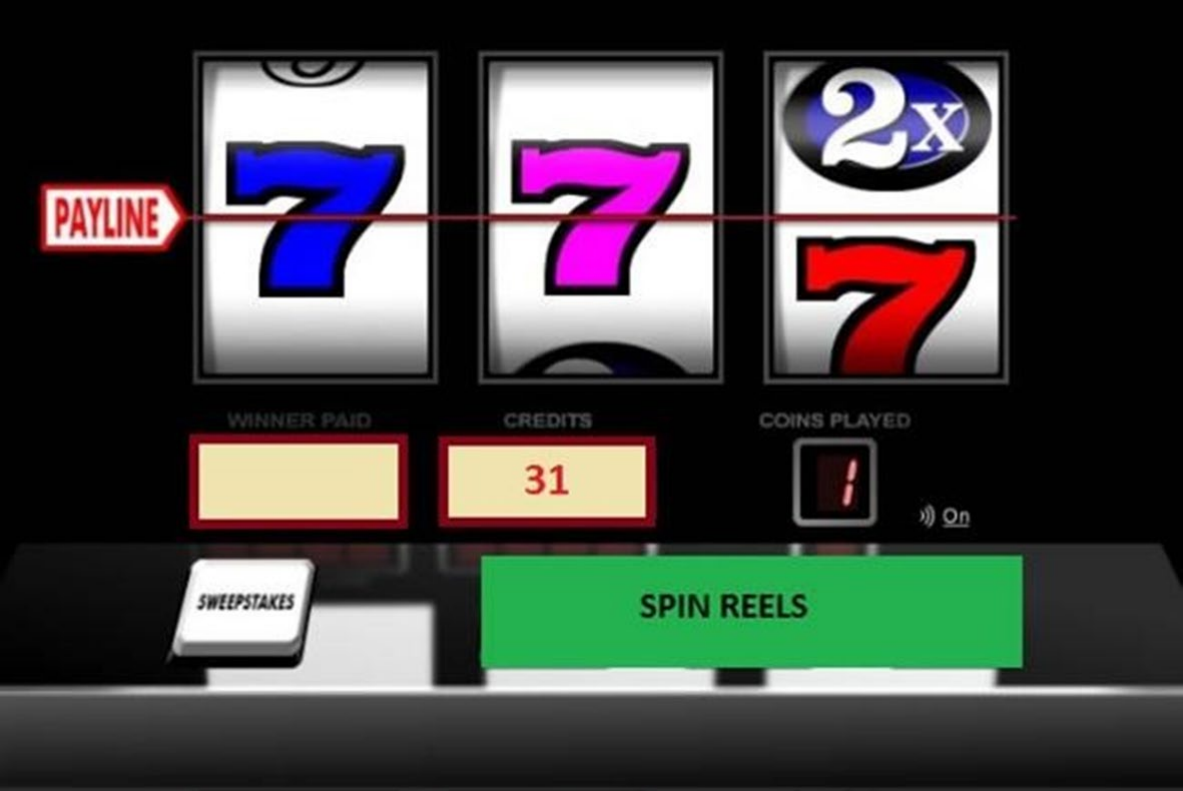
Image taken from the electronic slot machine simulator programme. A three-reeled slot machine simulator with a single pay line was designed using *PsychoPy* experiment builder. The machine is activated using the space bar on the participant’s keyboard when the visual display spin button changes from grey to either green or red (though participants are instructed to withhold responses when the button is red) (Color figure online)

**Fig. 2:**
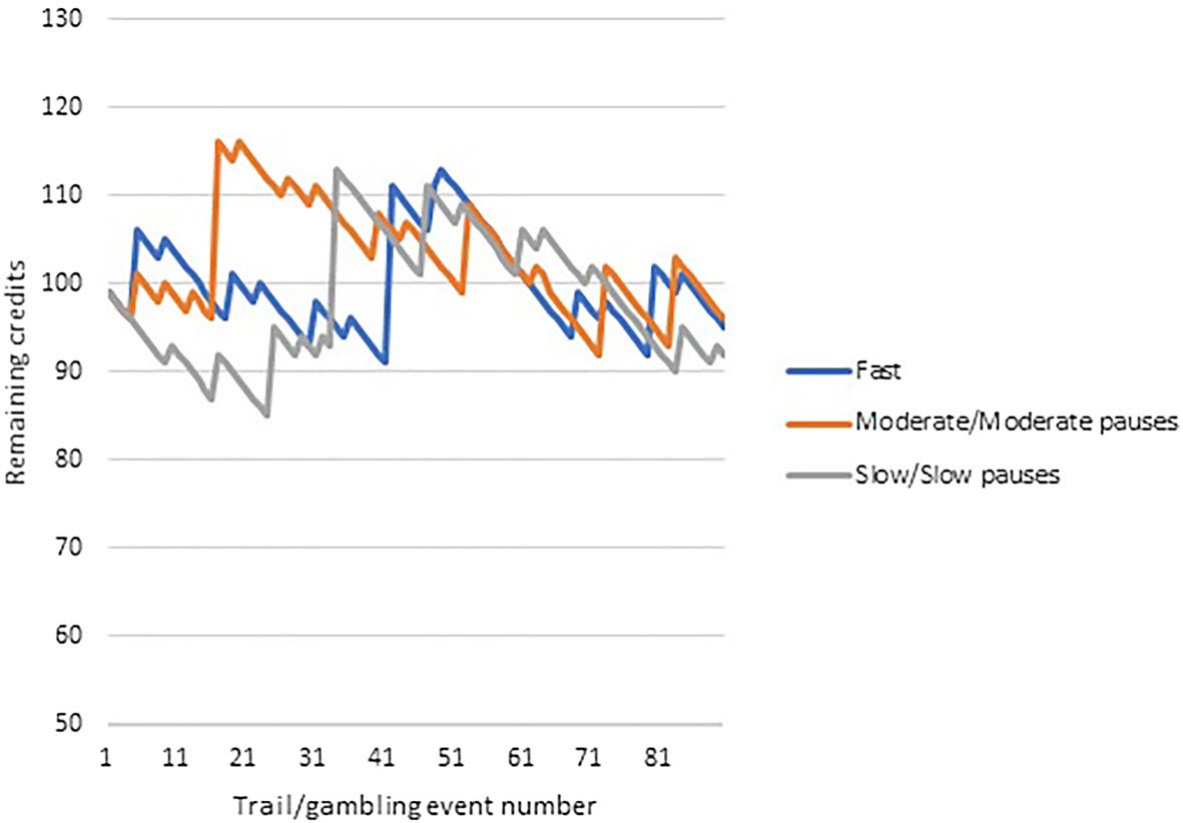
Series of wins and losses for each of the slot machine speed of play conditions. The moderate speed with pauses machine and slow speed with pauses machine had the same outcome series as the moderate and slow speed machines respectively but varied in the visual symbols presented on the reels on non-win trials. Participants start with 100 credits in each condition

A repeated-measures experiment was conducted to assess the impact of slot machine event frequency on motor response inhibition performance. An electronic slot machine simulator was designed using a combination of the graphical user interface and coding function available on *Psychopy* experiment builder (Peirce 2007) (see Fig. 1). The slot machine was a three-reeled design, with a single pay line, comprising five speed of play conditions: fast; moderate; and slow slot machine event frequencies (1.5 s, 3 s, and 4.5 s event frequencies respectively); moderate event frequency with a brief pause in play (fast spin of 1.5 s plus 1.5 s pause in play, totalling 3 s event frequency); and slow event frequency with brief pause in play (fast spin of 1.5 s plus 3 s pause in play, totalling 4.5 s event frequency). Each condition of the slot machine simulator had 90 trials (gambling events). Each slot machine condition was programmed to give the illusion of randomness. However, the slot machines were pre-programmed to control for volume, frequency, and range of wins, as well as number of near misses (see Clark et al. 2009). However, there was a 4% variance in payback percentages among the five conditions to ensure participants did not win or lose the exact same amount in every condition, and therefore, reinforcing the illusion of randomness (see Fig. 2). The slot machine pay-back percentages ranged from 92 to 96%. This variance was considered small and not able to produce a significant enough change in valence as a result of increased/decreased monetary wins/losses, and therefore, was not considered to represent a confounding variable.

A behavioural measure of response inhibition, in the form of a Go/No-go task (see Fig. 1), was built into the slot machine simulator, and immediately following each session of gambling, participants were given various psychometric scales to complete to assess subjective arousal, dissociation, valence, and perceived self-control. All scales were presented and completed using the *PsychoPy* experiment builder. Reaction time was also measured, and is a standard function in the experimental software.

#### Participants

A sample of 50 (36 male) non-problem regular gamblers were recruited from amusement arcades and sports teams in the Lincolnshire region of the UK. These areas were targeted during the recruitment process as they were identified as areas most likely to contain a high density of gamblers. All participants were classed as regular gamblers, defined for the purposes of this study as an individual who had gambled at least once per month over the past 12 months. Participant mean age was 29.88 years (SD = 9.13), with ages ranging from 19 to 58 years. A short screening questionnaire was administered to both ensure participants reported regular participation in gambling, as well as to ensure participants had never experienced problem gambling, nor was a current problem gambler. An affirmative answer on either count of problem gambling resulted in participants being excluded from taking part in the experiment. Consequently, two participants were excluded from participation following the initial screening because they reported having previously experienced problem gambling.

#### Behavioural Response Inhibition Task

The electronic slot machine simulator consisted of 90 trials (gambling events) per condition. The machine was activated by pressing the ‘spin button’ which was the spacebar on a standard keyboard. The spin button on the slot machine simulator visual display varied in colour from green to red, with green trials indicating participants could spin the machine and continue gambling, and red indicating that they needed to withhold their motor response. Response inhibition was therefore assessed with an ‘online’ behavioural Go/No-go task (embedded within the gambling simulator). The first 30 trials of each condition were all green ‘go’ responses, often referred to as a ‘training phase’ in classic response inhibition tasks (for a review, see Simmonds et al. 2008). The purpose of the first 30 trials all being ‘go’ trials was to allow any prepotent patterns of motor responses to develop. The remaining 60 trials in each condition consisted of a random 4:1 ratio of green ‘go’ to red ‘no-go’ trials.

#### Dissociation

Dissociative experience was assessed using a modified version of Jacobs’ (1988) four-item Dissociative Experience Scale (DES). The original scale was modified in two ways for the present study. First, the original four items were modified to ask participants to reflect on the gambling session they had just participated in, as opposed to gambling experience in general. For example, the question ‘When gambling, how often do you feel like you have been in a trance?’ was modified to read ‘Thinking back to the gambling session you have just completed, how often did you feel like you were in a trance?’ The second modification of the scale was the addition of a fifth item, asking participants about their perception of time during the gambling session, an item incorporated into previous experimental gambling research (see Gupta and Derevensky 1998; Blaszczynski et al. 2015a, b). All five items were self-report on a five-point Likert-scale, anchored at 1, ‘never’, and five, ‘all the time’. Midpoint of the scale, 3, indicated ‘occasionally’.

#### Subjective Arousal and Valence

Participant subjective levels of arousal and valence during each experimental condition were assessed using the Self-Assessment Manikin (SAM; Lang 1980). The SAM is a non-verbal pictorial assessment technique that directly assesses the pleasure and arousal associated with an individual’s affective reaction to a wide variety of stimuli. The SAM was chosen to assess valence and arousal because it is a method that has been demonstrated as an easy to administer, non-verbal method for quickly assessing the arousal and pleasure associated with an individual’s reaction to an event or stimuli. SAM scores measuring experience of arousal are highly correlated with scores obtained using the verbal and lengthier Semantic Differential Scale (Bradley and Lang 1994). They have also been used to assess emotional responses to a wide range of stimuli, including both pictures (e.g., Lang et al. 1993) and sounds (e.g., Bradley 1994), as well as being successfully administered across a range of clinical populations, as well as children and non-English speakers (Bradley and Lang 1994). Full body versions of the SAMs were used for both the valence and arousal scale (portrait-only versions are available for the valence scale), and both scales were presented in their nine-point scale versions.

#### Perceived Self-control

Participants’ perceived level of self-control was assessed using a single-item nine-point Likert scale questionnaire. Perceived self-control was assessed to ascertain to what extent participants felt they were exercising self-control during the various gambling conditions. Participants were asked, ‘To what extent do you feel you were in control of your actions during the last gambling session?’ Responses were anchored at 1, ‘no self-control’, and 9, ‘maximum self-control’. The midpoint of the scale, 5, indicated ‘moderate levels of self-control’. Perceived self-control was assessed using a separate question due to concerns that scores on this item could vary greatly because of subjective interpretation of the dominance item. For example, participants could interpret dominance as their perceived performance during gambling in terms of money won/lost, as opposed to the item’s intention of assessing control over the situation. Furthermore, the present study was concerned with how actual levels of motor control compared to perceived levels of motor control, and therefore, it was deemed more accurate to use an item that was explicitly clear which component of self-control participants should self-rate.

### Procedure

Each participant gambled on a three-reeled electronic slot machine simulator in the five aforementioned conditions. The purpose of providing a brief pause in play following the spinning of the reels was in line with the third aim of the study (i.e., to investigate if brief pauses in play allow a gambler to adaptively modulate their behaviour). It was hypothesised that providing a short pause following presentation of a gambling result will provide a refractory period to allow executive control systems to catch-up with actions that may be automatically stimulated by the provision of a new gambling event. Participants were provided with £20 to gamble with and were told that any money they had left at the end of the gambling session could be kept. The £20 was converted into 500 credits, and the credits were split equally among each of the five experimental conditions, meaning each participant had a starting credit total of 100 in each condition (£4). The order of the gambling conditions was counterbalanced using a Latin Squares method and participants were given a 5-min break in between each gambling condition.

**Fig. 3:**
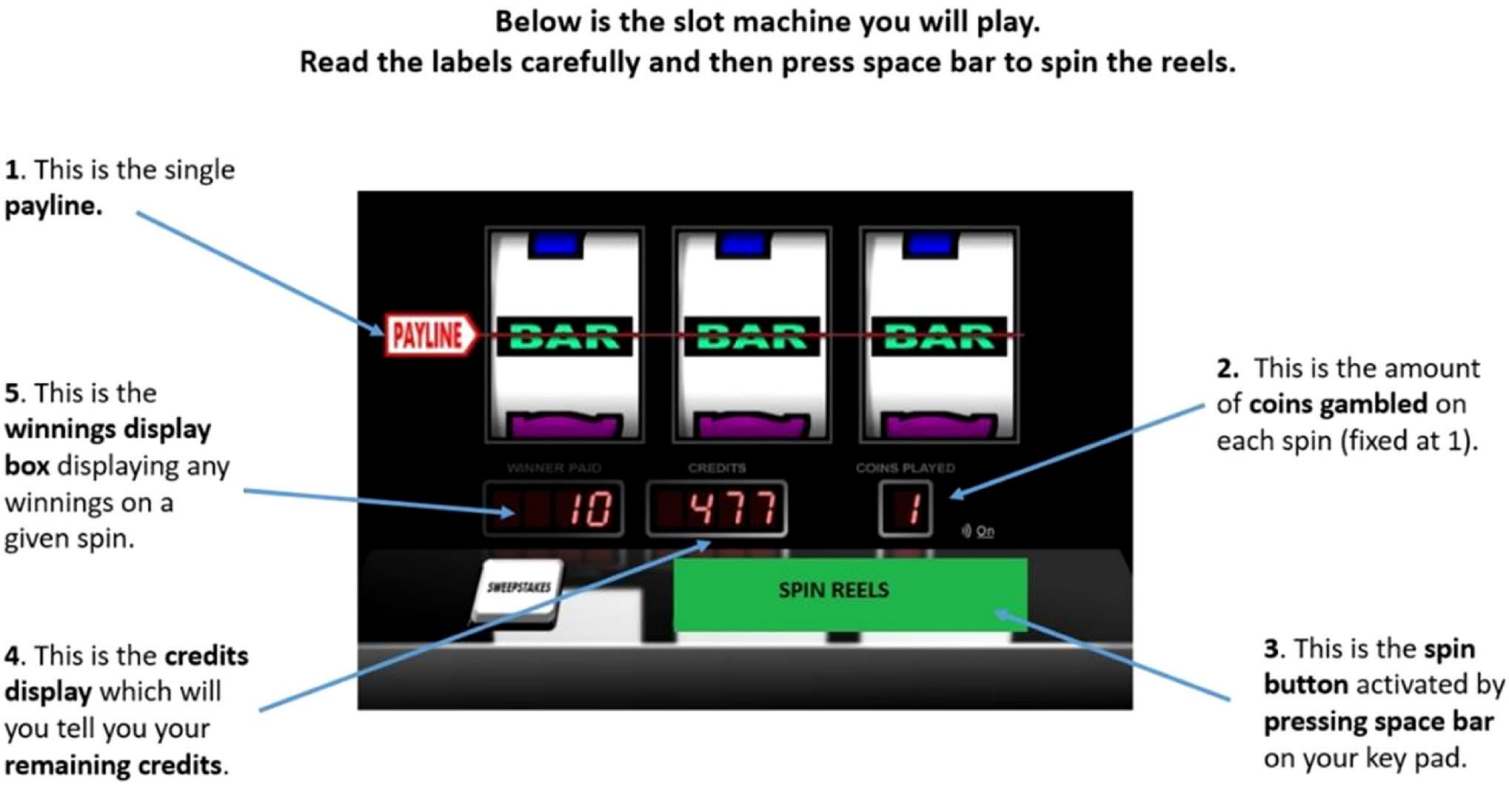
Slot machine instructions presented to participants during the tutorial prior to the gambling simulation

**Fig. 4:**
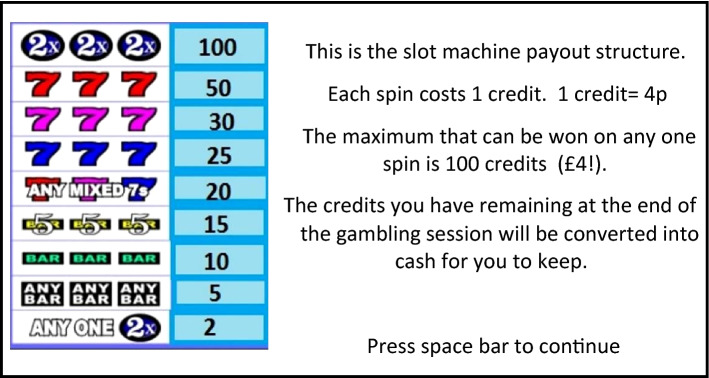
Slot machine pay-out structure presented to participants during the tutorial prior to the gambling simulation

Participants were given a tutorial in how to operate the slot machine and were informed of what each of the visual display features were, including the pay-line, credit balance, and win totals on winning spins (see Fig. 3). A pay-out structure was also shown to participants during the tutorial, showing how much money could be won for specific matching symbols (see Fig. 4). Participants were instructed to only operate the machine by pressing the spin button (space bar on standard computer keyboard) when the spin button on the visual display was green in colour, and instructed they must withhold from pressing the spin button when it was red in colour. The slot machine was programmed to spin automatically on no-go trials after a delay equivalent to one event frequency which was dependent on the speed of the slot machine. The first 30 trials of each slot machine condition were all ‘go’ trials, allowing potential response prepotency to develop, and the remaining 60 trials consisted of a 4:1 ratio of ‘go’ to ‘no-go’ trials. Following each gambling condition, participants were instructed to complete the arousal and valence SAM, the single-item self-control question, and the four-item DES in that order. All scales were completed on a computer immediately following the gambling simulation in each condition.

### Ethics

Before commencement of the study, the study was approved by the research team’s University Ethics Committee. The study protocol was designed in accordance with guidelines of the Declaration of Helsinki. Participants were fully briefed and instructed on how to complete all tasks prior to the beginning of the experiment and provided their informed consent to take part in the study. Participants were informed that all their data were confidential and anonymous.

## Results

### Response Inhibition Performance

The value for the dependent variable response inhibition performance was derived by calculating the percentage for which the gamblers were able to successfully withhold motor responses on no-go slot machine trials. Successfully withholding motor response on all 12 no-go trials therefore returned a response inhibition performance score of 100%.

**Fig. 5:**
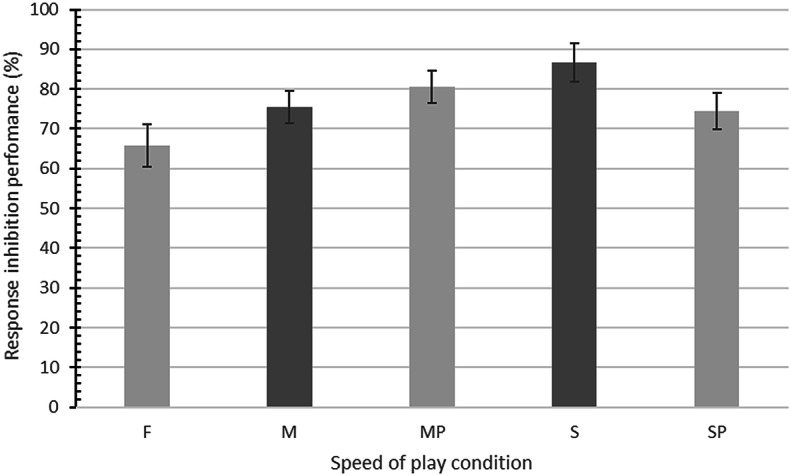
Mean percentage of successfully inhibited motor responses in the fast (F), moderate (M), moderate with pauses (MP), slow (S), and slow with pauses (SP) speed of play conditions. Error bars represent 95% confidence intervals

Mean response inhibition performance in the fast speed condition (1.5 s event frequency) was 65.8% (SD = 18.54), 75.50% (SD = 14.03) in the moderate speed condition (3 s event frequency), and 86.67% (SD = 16.84) in the slow speed condition (4.5 s event frequency), indicating a trend towards increased impulsivity as speed of play increased. Mean response inhibition performance in the moderate speed condition with a brief pause in play was 80.50% (SD = 14.35), a 5% increase compared to the moderate speed condition with no pause in play. Performance in the slow speed condition with a brief pause in play was 74.50% (SD = 16.01), a 12% reduction compared to the slow speed condition with no pause in play. All response inhibition performance means and standard deviations can be found in Table 1 and are presented in Fig. 5.

**Table 1:** Mean (SD) dependent variable scores and ANOVA *p* values across speed of play conditions

Speed condition	Dependent variable					
	Response inhibition (%)	Reaction Time (s)	Dissociation total (5–25)	Arousal (1–9)	Valence (1–9)	Perceived self-control (1–9)
Fast	65.80 (18.54)	.61 (.23)	6.48 (1.34)	6.66 (1.39)	5.66 (1.19)	6.78 (.89)
Moderate	75.50 (14.03)	.72 (.21)	7.04 (1.52)	5.56 (1.33)	4.46 (1.18)	6.82 (1.10)
Moderate with pauses	80.50 (14.35)	.74 (.19)	7.14 (1.85)	4.92 (1.18)	4.26 (1.17)	6.86 (1.13)
Slow	86.67 (16.84)	.84 (.18)	9.76 (2.92)	3.64 (1.05)	3.38 (1.12)	6.96 (1.12)
Slow with pauses	74.50 (16.01)	.69 (.19)	8.10 (2.55)	5.36 (1.76)	2.76 (1.08)	6.58 (1.31)
ANOVA *p* value	< .001	< .001	< .001	< .001	< .001	= .086

A one-way repeated-measures ANOVA showed the differences in means were statistically significant, F(4245) = 11.57, *p *< .001, *η*^*2*^= .159. Bonferroni pairwise comparisons indicated that performance in the fast condition was significantly worse when compared to the moderate speed (*p *< .001, *d *= .59), moderate speed with pauses (*p *< .001, *d *= .88) and slow speed conditions (*p *< .001, *d *= 1.18). Performance in the moderate speed condition was also significantly worse than performance in the slow speed condition (*p *= .003, *d *= .72).

The Bonferroni pairwise comparisons also showed a non-significant difference between response inhibition performance at moderate speeds of play when compared to performance at moderate speeds with a brief pause in play (*p *= .99, *d *= .35). Conversely, pair-wise comparisons showed a significant difference between performance at slow speeds of play compared to performance at slow speeds of play with brief pauses in play (*p* < .001, *d *= .74). However, results indicate that performance was impaired with the inclusion of the pauses at slow speeds of play.

### Overall Reaction Time

Mean reaction time values were derived from measuring the average time between the start of the opportunity to gamble on a gambling trial and participants pressing the spin button on the slot machine simulator, measured in seconds. Mean reaction time for the fast speed of play condition was .61 s (SD = .23), .72 s (SD = .21) for the moderate speed condition, and .84 s (SD = .18) for the slow speed condition, indicating a trend towards faster response times as speed of play increased. Mean reaction time for the moderate speed with brief pauses in play condition was .74 s (SD = .19), indicating a marginal slowing of reaction time when compared to moderate speeds of play with no pause in play. Mean reaction time for the slow speed condition with brief pauses in play was .69 s (SD = .19), indicating an approximate 17% decreases in reaction time when compared to slow speeds of play without pauses in play. All reaction times and standard deviations can be found in Table 1.

A one-way repeated-measures ANOVA showed the differences between mean reaction times across conditions were statistically significant, F(4196) = 9.82, *p *< .001, *η*^*2*^= .138. Bonferroni pairwise comparisons showed that reaction time in the fast speed condition was significantly faster compared to the moderate speed (*p *< .001, *d *= .58) and slow speed condition (*p *< .001, *d *= 1.35), and significantly faster in the moderate speed condition compared to the slow speed condition (*p *= .001, *d *= .63)

The Bonferroni pairwise comparisons also showed that the mean reaction times in the moderate speed condition did not differ to a statistically significant level when compared to the moderate speed with pauses in play condition (p = .99, *d *= .10). However, pairwise comparisons did show that mean reaction time in the slow speed condition was significantly slower when compared to the slow speed with pauses in play condition (*p *= .02, *d *= .83), counterintuitively indicating faster reaction times were recorded as a result of providing pauses in play.

#### Dissociation

Overall dissociation scores for each participant were derived by summing the scores for each of the five-items on the DES. As ratings on each item could be made on a 1–5 scoring system, the minimum and maximum overall dissociation score was 5 and 25 respectively. Mean dissociation scores for the fast speed of play condition were 6.48 (SD = 1.34), 7.04 (SD = 1.52) for the moderate speed condition, and 9.76 (SD = 2.92) for the slow speed condition, indicating a trend towards lower levels of dissociation as speed of play increased. Of note, dissociation scores overall across conditions were low because even in the slow speed condition where dissociation was highest, mean scores here were only approximately equivalent to a rating of ‘rarely’ for all items. The mean dissociation score in the moderate speed with pauses in play condition was 7.14 (SD = 1.85), a negligible increase when compared to moderate speeds without pauses in play. The mean dissociation score for the slow speed with pauses in play condition was 8.10 (SD = 2.55), a 17% decrease when compared to slow speeds without pauses in play. All dissociation scores and standard deviation can be found in Table 1.

A one-way repeated-measures ANOVA showed the differences in mean dissociation scores across conditions were statistically significant, F(4196) = 18.32, *p *< .001, *η*^*2*^= .23. Bonferroni pairwise comparisons showed that dissociation levels in the fast speed condition were significantly lower when compared to the moderate (*p *< .001, *d *= .39) and slow speed condition (*p *< .001, *d *= 1.44), and significantly lower in the moderate speed condition compared to the slow speed condition (*p *< .001, *d *= 1.17).

Pairwise comparisons also showed that dissociation scores in the moderate speed with pauses in play condition did not differ significantly when compared to moderate speeds without pauses in play (*p *= .99, *d *= .06). However, dissociation scores in the slow speed with pauses in play condition were significantly lower when compared to slow speeds without pauses in play (*p *= .001, *d *= .61). These results indicate that brief pauses in play reduced dissociation levels, but only at slow game speeds.

### Arousal

Mean arousal score, rated on a single-item scale ranging from 1 to 9, for the fast speed of play condition was 6.66 (SD = 1.39), 5.56 (SD = 1.33) for the moderate speed condition, and 3.64 (SD = 1.05) for the slow speed condition, indicating a trend towards increased levels of arousal as speed of play increased. Mean arousal score for the moderate speed of play with pauses in play condition was 4.92 (SD = 1.18), an approximate 12% decrease when compared to moderate speeds without pauses in play. Mean arousal score in the slow speed with pauses in play condition was 5.36 (1.76), a 47% increase when compared to slow speeds without pauses in play. All mean arousal scores and standard deviations can be found in Table 1.

A one-way repeated-measures ANOVA showed the differences in mean arousal scores across conditions were significant, F(4196) = 51.09, *p *< .001, *η*^*2*^= .35. Bonferroni pairwise comparisons showed that arousal scores in the fast speed condition were significantly higher when compared to the moderate (*p* < .001, *d *= .81) and slow speed condition (*p *< .001, *d *= 2.45), and significantly higher at moderate speeds compared to slow speeds (*p *< .001, *d *= 1.60).

Pairwise comparisons also showed that mean arousal score was significantly lower in the moderate speed with pauses in play condition compared to the moderate speed without pauses in play condition (*p *= .004, *d *= .51). However, conversely, arousal levels were significantly higher in the slow speed with pauses in play condition compared to slow speed without pauses in play condition (*p *< .001, *d *= 1.19). Taken together these findings suggest the impact of brief pauses in play on subjective arousal interact with speed of play because the directional change in arousal as a result of pauses in play is dependent upon game speed.

### Valence

Mean valence score, rated on a single-item scale ranging from 1 to 9, for the fast speed of play condition was 5.66 (SD = 1.19), 4.46 (SD = 1.18) for the moderate speed condition, and 3.38 (SD = 1.12) for the slow speed condition, indicating a trend towards increased positive valence as speed of play increased. Mean valence score for the moderate speed with pauses in play condition was 4.26 (SD = 1.17), an approximate 4% decrease when compared to valence ratings for moderate speeds without pauses in play. Mean valence score for the slow speed with pauses in play condition was 2.76 (SD = 1.08), an approximate 18% reduction when compared to slow speeds without pauses in play. All mean valence scores and standard deviations can be found in Table 1.

A one-way repeated-measure ANOVA showed the differences in mean valence scores were significant, F(4196) = 86.04, *p *< .001, *η*^*2*^= .43. Bonferroni pairwise comparisons showed that mean valence score in the fast speed condition was significantly higher compared to the moderate speed (*p *< .001, *d *= 1.01) and slow speed condition (*p* < .001, *d *= 1.97), and significantly higher at moderate speeds compared to slow speeds (*p *< .001, *d *= .94).

Pairwise comparisons also showed that mean valence score in the moderate speed with pauses in play condition did not differ significantly from mean valence score in the moderate speed without pauses in play condition (*p* = .96, *d *= .17). However, mean valence score was significantly lower in the slow speed with pauses in play condition compared to the slow speed without pauses in play condition (*p *< .001, *d *= .56), indicating that pauses in play only significantly reduced valence ratings when applied at slow speeds of play.

### Perceived Self-control

During statistical assumption testing, one extreme outlier was found in every speed of play condition for the perceived self-control variable. Upon closer inspection of these outliers, it was found that the same participant provided all of these data points and thus, their data for the self-control variable was removed from further analysis. Mean perceived self-control score, rated on a single-item scale ranging from 1 to 9, for the fast speed of play condition was 6.78 (SD = .89), 6.82 (SD = 1.10) for the moderate speed condition, and 6.96 (SD = 1.12) for the slow speed condition, indicating a negligible change in perceived self-control ratings as a result of speed of play. Mean self-control score for the moderate speed with pauses in play condition was 6.86 (SD = 1.13), a negligible increase when compared to moderate speeds without pauses in play. Mean self-control score for the slow speed with pauses in play condition was 6.58 (SD = 1.31), an approximate 5% decrease when compared to self-control ratings for the slow speed without pauses in play condition. All mean perceived self-control scores and standard deviations can be found in Table 1. A one-way repeated-measures ANOVA showed the differences in mean perceived self-control scores across conditions failed to reach statistical significance, F(4195) = 2.23, *p *= .086, *η*^*2*^= .01.

### Response Modulation

To assess if participants were able to modify their behaviour in order to facilitate response inhibition performance (proactive inhibition), participant reaction time was measured during the ‘training’ phase of each condition, that is, the first 30 trials in each condition in which only ‘go’ trials are presented, and compared to participant mean reaction times from the onset of the first ‘no-go’ trial to the end of each condition. A statistically significant slowing of reaction time is thus interpreted as adaptive behavioural modulation because it represents a proactive effort to avoid commission errors on the embedded Go/No-go task.

**Fig. 6:**
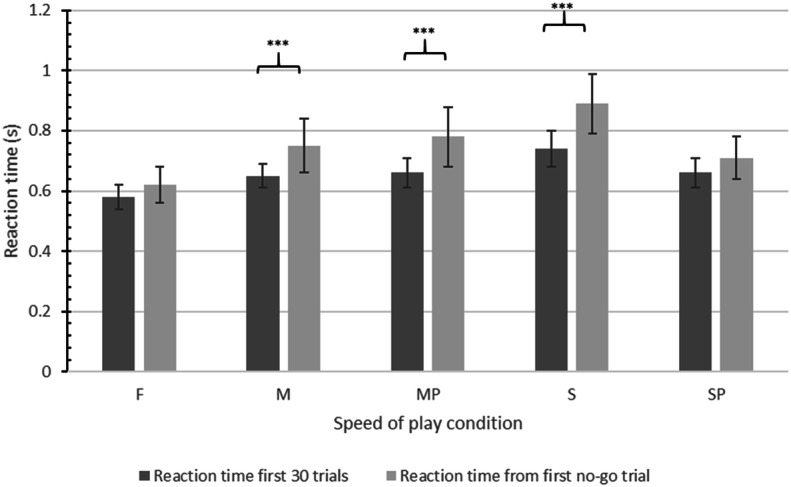
Mean reaction time in seconds (s) for first 30 trials and from the onset of the first no-go trial. Error bars depict 95% confidence intervals. ***p < .001

Paired-sample *t* tests showed evidence for this behavioural modulation at moderate speeds, slow speeds, and moderate speeds with pauses in play, but was not demonstrated at fast speeds or slow speeds with pauses in play. In the fast condition, mean reaction time for the first 30 trials was .58 s (SD = .16) compared to a mean reaction time of .62 s (SD = .20) from the onset of the first ‘no-go’ trial, a slowing of reaction time that just failed to reach statistical significance, *t*(49) = 1.98, *p *= .054, *d *= .22. In the moderate speed condition, mean reaction time for the first 30 trials was .65 s (SD = .15) compared to .75 s (SD = .24) from the onset of the first ‘no-go’ trial, a slowing of reaction time that reached statistical significance, *t*(49) = 4.23, *p *< .001, *d *= .49. In the moderate speed with pauses in play condition, mean reaction time for the first 30 trials was .66 s (SD = .17) compared to .78 s (SD = .23) from the onset of the first no-go trial, a slowing of reaction time that reached statistical significance, *t*(49) = 5.42, *p *< .001, *d *= .59. In the slow speed condition, mean reaction time for the first 30 trials was .74 s (SD = .19) compared to .89 s (SD = .22) from the onset of the first ‘no-go’ trial, a slowing of reaction time that reached statistical significance, *t*(49) = 6.03, *p *< .001, *d *= .73. Finally, in the slow speed with pauses in play condition, mean reaction time for the first 30 trials was .66 s (SD = .18) compared to .71 s (SD = .18) from the onset of the first ‘no-go’ trial, a slowing of reaction time that failed to reach statistical significance, *t*(49) = 1.89, *p *= .065, *d *= .28. The evidence therefore suggests that at fast speeds of play, gamblers were impaired in their ability to modulate behaviour adaptively. Table 2 and Fig. 6 summarises the response modulation findings.

**Table 2:** Mean (SD) participant response times to gambling stimuli for the first 30 trials compared to response times to gambling stimuli from the onset of the first no-go trial to the end of the condition

Speed condition	Mean reaction times (RT)		
	Mean RT for first 30 trials (s)	Mean RT from onset of first no-go trial (s)	t-test *p* value
Fast	.58 (.16)	.62 (.20)	= .054
Moderate	.65 (.15)	.75 (.24)	< .001
Moderate with pauses	.66 (.17)	.78 (.23)	< .001
Slow	.74 (.19)	.89 (.22)	< .001
Slow with pauses	.66 (.18)	.71 (.18)	= .065

*Note* Whilst adaptive response modulation was found in both the moderate speed and moderate speed with pauses conditions, additional analysis showed there was no statistically significant degree of response slowing between these two conditions, indicating no additional benefits stemming from the inclusion of pauses in play

The average slowing of reaction time upon the onset of ‘no-go’ trials was .10 s (SD = .13) in the moderate speed without pauses in play condition, and .12 s (SD = .10) in the moderate speed with pauses condition. While these means suggest a greater proportion of behavioural modulation took place with the inclusion of brief pauses in play, a paired-sample *t* test showed the difference in means failed to reach statistical significance, *t*(49) = .236, *p *= .815, *d *= .17, indicating the pauses in play had no additional advantage to response modulation at moderate speeds of play.

### Regression Findings

To investigate the psychological factors that predict impaired response inhibition performance, a series of multiple regression analysis were conducted. Multiple regression analysis was conducted on each speed of play condition separately, given both the theoretical rationale and empirical analysis conducted thus far, both of which provide a sound premise for an interaction effect between different speeds of play and the factors which impair response inhibition performance. This approach runs contrary to collapsing all data into a single condition to provide a single model of the factors that predict response inhibition performance, because this overlooks all interaction effects and ignores the differential psychological factors that different speeds of play impact. For all conditions, the variables of arousal, dissociation, valence, and reaction time were entered into the regression model as predictor variables using the entry method, and response inhibition performance was the outcome variable.

In the fast speed of play condition, arousal, and reaction time were variables predictive of response inhibition performance. The results of the regression indicated that arousal and reaction time accounted for 40.1% of the variance explained by the model (${\text{R}}_{\text{adjusted}}^{2}$ = .401, F(1,49) = 19.48, *MSE *= 87.30, *p *< .001). Arousal was a significant negative predictor of response inhibition performance in the fast speed condition (β = − .558, *p *< .001), and reaction time was a significant positive predictor (β = 257, *p *= .023).

At a moderate speed of play, arousal and dissociation were both found to be predictors of response inhibition performance. The results of the regression indicated that the two predictors (i.e., arousal and dissociation), accounted for 39.5% of the variance explained by the model (${\text{R}}_{\text{adjusted}}^{2}$ = .395, F(1,49) = 16.99, *MSE *= 119.04, *p *< .001). Arousal level was a significant negative predictor of response inhibition performance in the moderate speed condition (β = −  .427, *p *< .001), as were levels of dissociation (β = − .348, *p *= .033).

At a moderate speed of play with the inclusion of brief pauses in play, arousal and reaction time were both found to be predictors of response inhibition performance. The results of the regression indicated that the two predictors (i.e., arousal and reaction time), accounted for 43.9% of the variance explained by the model (${\text{R}}_{\text{adjusted}}^{2}$ = .439, F(1,49) = 20.15, *MSE *= 115.52, *p *< .001). Arousal was found to be a significant negative predictor of response inhibition performance at moderate speeds with pauses in play (β = − .574, *p *< .001), whereas reaction time was found to be a significant positive predictor of response inhibition performance (β = .263, *p *= .02).

At a slow speed of play, level of dissociation was found to be a predictor of response inhibition performance. The results of the regression indicated that dissociation accounted for 39.7% of the variance explained by the model (${\text{R}}_{\text{adjusted}}^{2}$ = .397, F(1,49) = 18.88, *MSE *= 152.39, *p *< .001). The level of dissociation was found to be a significant negative predictor of response inhibition performance at slow speeds of play (β = − .568, *p *< .001).

At a slow speed of play with the inclusion of brief pauses in play, arousal and dissociation were both found to be predictors of response inhibition performance. The results of the regression indicated that the two predictors (i.e., arousal and dissociation), accounted for 36.8% of the variance explained by the model (${\text{R}}_{\text{adjusted}}^{2}$ = .368, F(1,49) = 15.26, *MSE *= 161.99, *p *< .001). Arousal was found to be a significant negative predictor of response inhibition performance at slow speeds with pauses in play (β = − .509, *p *< .001), as was level of dissociation (β = − .445, *p *< .001). Table 3 below provide a summary of the standardised and unstandardised beta coefficients for all speed of play conditions.

**Table 3:** Summary of multiple regression model coefficients for all speed of play conditions

	Fast speed			Moderate speed			Moderate speed with pauses			Slow speed			Slow speed with pauses		
Variable	*B*	*SE B*	*β*	*B*	*SE B*	*β*	*B*	*SE B*	*β*	*B*	*SE B*	*β*	*B*	*SE B*	*β*
Arousal	− 11.53	.96	− .56***	− 5.90	1.19	− .43***	− 7.00	1.34	− .57***				− 4.63	1.04	− .51***
Dissociation				− 2.28	1.04	− .35*				− 2.58	.62	− .57***	− 2.80	.72	− .45***
Valence															
Reaction time	12.85	8.77	.26*				19.45	8.11	.26*						
*R*^*2*^		.40			.40			.44			.40			.37	
*F*		19.48			16.99			20.15			18.88			15.26	

*p < .05; **p < .01; ***p < .001

## Discussion

In support of H1, empirical evidence demonstrating that response inhibition performance among a sample of regular non-problem gamblers was significantly impaired at faster gambling speeds of play. The percentage of successfully withheld motor responses during the slot machine gambling simulation fell to 65.8% at fast speeds of play, compared to 75.5% at moderate speeds, and 86.7% at slow speeds. Subjective levels of arousal were also significantly increased during fast speeds of play, supporting H2 and the notion that games with higher event frequencies are more arousing for gamblers. Furthermore, in partial support of H3, subjective levels of arousal were found to be a significant and negative predictor of response inhibition performance during fast speeds of play, as well as moderate speeds of play. However, at the slowest speed of play, arousal was no longer a significant predictor of response inhibition performance, where the level of dissociation was the dominant predictive factor. These findings provide insight into how the psychological factors that predict response inhibition performance during gambling interact with game speed and suggests two routes to impaired response inhibition within a gambling context—an arousal route and a dissociation route. Finally, there was no support for H4, because brief pauses in play did not facilitate response inhibition performance and there was no evidence for brief pauses in play enhancing response modulation via proactive motor control. On the contrary, perverse effects were found when including pauses in play at slow speeds. At slow speeds, the inclusion of brief pauses in play had a significant and negative impact on response inhibition performance, where on average, a 12% reduction in response inhibition performance was found when compared to slow speeds without pauses in play.

### Valence

Valence ratings indicate that as the speed of play increased the enjoyment of the game also increased, consistent with the general findings from a systematic review of speed of play in gambling conducted by Harris and Griffiths (2018). Valence ratings also showed that brief pauses in play at a moderate speed of play did not detract from the enjoyment of the game when compared to moderate speeds without pauses, although this finding appears to be in vein, because pauses were not effective in facilitating response inhibition. However, the inclusion of pauses in play at slow speeds of play significantly reduced enjoyment of the game when compared to slow speeds without pauses. This, along with the finding that response inhibition was impaired and arousal was increased at slow speeds with pauses compared to slow speeds without pauses, suggests a frustration effect and that participants may have become inpatient due to the increased time delay between completion of the reel spin and the next gambling event. This is consistent with previous research demonstrating an association between imposed breaks in play and gambling cravings, an effect that was mediated by subjective negative arousal (Blaszczynski et al. 2015a, b). Although the imposed breaks in play in Blaszczynski et al. (2015a, b) research equated to breaks of several minutes, compared to several pauses of only a few seconds in the present experiment, it may be argued that placing a barrier between a gambler and gambling, in this case in the form of pauses in play, may give rise to an aversive state that is detrimental to self-control. Such a finding is consistent with the conceptual model of behavioural completion proposed by McConaghy (1980) and Tiffany (1990), that states imposing barriers on gamblers in an approach state will result in negative affective states and increased urges to gamble. These increased urges may give rise to approach behaviours and impulsive action in pursuit of gambling and may explain the reduced inhibition performance demonstrated at slow speeds with pauses in play compared to slow speeds without pauses.

### Perceived Self-control

Perceived self-control ratings were consistently high across all conditions and did not differ statistically between conditions. These subjective self-control findings contradict the objective results obtained from the behavioural response inhibition task. The behavioural results showed a clear reduction in response inhibition performance as speed of play increased, and yet participants did not fluctuate in their perceived levels of self-control. Two explanations for this disparity in results are offered. First, it possible that the reduction in inhibitory control observed at fast speeds of play occurred subconsciously due to high levels of engagement with the gambling simulation. An alternative explanation might be that what a gambler views as self-control does not constitute the ability to withhold motor responses and may consist of behavioural markers that are more superficial, such as time and money spent gambling, factors controlled for in this experiment. Both of these explanations point to a lack of awareness of the role of response inhibition in self-control, either because the effect of speed of play on motor control is happening subconsciously, or due to the gambler’s lack of awareness of the important role of response inhibition in self-control. Changing the nature of the question given to the participants designed to assess perceived self-control may shed further light on this in future research. For example, participants could be asked more simply to state how well they think they did on the response inhibition task, and then compare this to actual performance to test the conscious or subconscious theories proposed here.

The two arguments represent an important distinction with different implications for gambling harm-minimisation approaches. If increased speeds of play result in sub-conscious response inhibition deficits, then it might be fruitful for harm-minimisation approaches (e.g., pop-up responsible gambling messaging), to draw attention to indicators of reduced inhibition performance, including rapid response styles and failure to withhold motor responses. This could also take the form of motor feedback, whereby machines could provide an aversive audio tone if the gamble/spin buttons are being pre-emptively pressed before an appropriate event frequency duration. If the issue is a lack of appreciation by gamblers of the role of response inhibition in self-control, then effort might be best placed with educational approaches that highlight the association between poor response inhibition and disordered gambling.

### Reaction Time

Unlike typical response inhibition tasks, participants in this experiment were not instructed to respond as fast and accurately as possible to allow participants to behave more naturally. Despite this, the time from the onset of a new gambling event to the participant executing a motor response to gamble was under one second in all conditions, suggesting slot machine gambling in general is associated with fast motor response speeds. Reaction time differed to a significant degree across speed of play conditions, with a trend towards faster reaction times as the speed of the game increased. One possible explanation for this finding is that behavioural synchronisation was occurring in response to the speed of the game. This phenomenon may be likened to examples outside of a gambling context where behaviour can be synchronised with environmental cues (Codrons et al. 2014). A prominent example is that individuals are seen to walk faster in urban environments when exposed to higher tempo music (Franek et al. 2014). The structural gambling feature of speed therefore appears to have the ability to influence behaviour in similar way, which is problematic given than faster motor reaction times were predictive of poor response inhibition performance in the present experiment. If faster games are reducing reaction time, time spent making a decision is also reduced. Consistent with the speed-accuracy trade-off literature (see e.g. Duckworth et al. 2018), this is likely to result in more erroneous and maladaptive responses being made.

### Response Modulation

One adaptive and proactive strategy to avoid erroneous responses on ‘no-go’ trials would be to modulate responses in favour of slower overall response speeds upon the onset of ‘no-go’ trials. This would provide increased time to process ‘go/no-go’ cues and increase the likelihood of correct responses being executed. Evidence for this adaptive response modulation was found in the moderate speed, moderate speed with pauses, and slow speed conditions, where overall participant reaction times increased (slowed) upon the onset of ‘no-go’ trials. However, this was not demonstrated in the fast speed or slow speed with pauses conditions, where reaction times did not change between the training phases and remaining 60 trials containing ‘no-go’ cues. This arguably represents reduced or impaired supervision by the executive system (see Verbruggen et al. 2012) in these gambling conditions. Of note, arousal was highest in these two conditions, which supports the association between arousal and response modulation found in previous studies that have also suggested that arousal has a detrimental impact on proactive motor control (Berkman et al. 2014; Verbruggen and De Houwer 2007). This suggests a potential causal pathway in the relationship between gambling speed and lack of proactive motor control.

### Arousal, Dissociation, and Reaction Time as Predictors of Response Inhibition

The finding that a gambler’s level of arousal was a significant and negative predictor of response inhibition performance is consistent with previous research outside of gambling that demonstrates increases in arousal result in poorer inhibition performance (e.g., Nieuwenhuis and de Kleijn 2013). Although reaction time within the fast speed condition was also significant predictor of response inhibition, arousal was the by far the dominant predictor of response inhibition performance at fast speeds of play. Subsequent analysis for mediation regression using the four steps approach proposed by Baron and Kenny (1986) showed that the effect of these two predictors were independent, because there was a lack of evidence to suggest that reaction time mediated the effect of arousal on response inhibition. This is contrary to theoretical and empirical accounts stating that increased arousal leads to a state of readiness to respond, where increased arousal lowers response thresholds and biases go and stop processes in favour of executing an action (see Logan and Cowan 1984; Nieuwenhuis and de Kleijn 2013; Posner and Petersen 1990).

Counterintuitively, poorer response inhibition performance is typically associated with *slower* reaction times in problem and pathological gambling groups, explained as a problem of response conflict resolution and disorganised stimulus–response schematics amongst these clinical groups (Kertzman et al. 2011; Odlaug et al. 2011; van Holst et al. 2010). This highlights that there may be qualitative differences between disordered gambling groups and healthy regular gamblers, particularly given the finding here that in fact *faster* reaction times were predictive of poorer inhibition performance. The findings here, in relation to reaction time as a predictor of response inhibition performance, are more consistent with speed-accuracy trade-off accounts of performance, where decisions that are made more slowly have higher accuracy compared to fast decisions that are associated with higher error rates (Chittka et al. 2009; Duckworth et al. 2018). It is less clear whether these differences represent a progression in the symptomology of disordered gambling, or whether predispositional and/or comorbidity factors account for the differences seen in problem gamblers in response inhibition tasks. The evidence here suggests that for regular non-problem gamblers, faster gambling speeds of play lead to elevated levels of arousal which in turn leads to impulsive response styles detrimental to executive control, but this process appears independent of faster reaction times. Therefore, results here may be supportive of more recent theoretical explanations of the effect of arousal on response inhibition. For example. Verbruggen et al. (2014) propose that perceptual processing, which is susceptible to the effects of arousal, may represent a single underlying process that plays a key role in behavioural inhibition.

Whilst this view would predict inhibition performance can be enhanced by increased arousal if this is also met with task-relevant information being made salient, facilitating processing of essential information, it also conversely predicts that if increases in arousal are accompanied with distracting information, then arousal may impair inhibition performance. If the act of gambling is considered the primary task in this simulation, then the increased arousal may have led to increased processing of gambling stimuli at the expense of efficient processing of ‘go’ and ‘no-go’ cues, resulting in poorer inhibition performance. In this account, gamblers may be inefficiently processing ‘no-go’ cues because arousal increases their allocated attention towards gambling stimuli in the visual field.

As the speed of play decreased, the relative predictive strength of arousal in response inhibition performance decreased. At moderate speeds, whilst arousal remained a predictor of response inhibition performance, overall levels of dissociation also became a significant and negative predictor. At slow speeds, arousal was no longer a significant predictor, and yet the predictive strength of dissociation on response inhibition performance increased. This demonstrates an interaction effect between speed of play and the psychological variables predictive of response inhibition performance. However, an alternative explanation is that the dissociation predictive of response inhibition at slower speeds of play may not be a product of the speed of the game but a result of the increased time spent gambling during this condition. The number of gambling trials were controlled across all condition, but as event frequency was experimentally manipulated, this naturally led to changes in the time spent gambling across conditions. As event frequency was three times slower in the slow condition compared to the fast condition, the gambling session was approximately three times longer at slow speeds of play, providing more opportunity for dissociative experience to develop. To test this time-based rather than speed-based dissociation explanation, further experimental research would be required, controlling for gambling session duration whilst simultaneously manipulating speed of play.

At fast speeds, arousal may be maladaptive in adjusting the perceptual processing of ‘go’ and ‘no-go’ cues, whereas slower speeds give rise to increases in dissociative experience predictive of poorer response inhibition performance. As dissociative experiences within a gambling context have been described as a reduced state of awareness or a period of ‘zoning out’ (Allcock et al. 2006), it is argued that attentional mechanisms may again drive this effect found here. A reduced attentional awareness of ‘no-go’ cues could account for the predictive value of dissociation in response inhibition performance found in moderate and slower game speeds. Perceptual processing may therefore be affected by separate processes. The arousal account found at fast speeds of play may explain reduced inhibition performance via enhanced processing of gambling stimuli and reduced attention towards ‘no-go’ cues. Dissociation can account for this impaired perceptual processing explanation via an overall reduced level of awareness during the gambling simulation, which also includes impaired processing of ‘go’ and ‘no-go’ cues.

If adjusted perceptual processing represents an underlying causal mechanism between arousal/dissociation and response inhibition, then it may be useful to conceptualise these distinctive processes as representing a state of maladaptive ‘zoning in’ and ‘zoning out’. In this instance, increased arousal resulting from fast speeds of play predicts poorer response inhibition because attention is focused on gambling at the expense of other important environmental cues, such as the ‘no-go. cues in this simulation. Conversely, the increased dissociation experienced at slower speeds of play results in an overall reduced amount of conscious perceptual processing of stimuli in the visual field, including reduced ability to process the ‘no-go’ cues and therefore withhold motor responses. However, this would suggest a dissociative relationship between arousal and levels of dissociation. Problematic for this position are theoretical accounts describing dissociation as an epiphenomenon of increased levels of arousal (Allcock et al. 2006). It may be the case that high arousal is a precursor for dissociative experience at the pathological level, such as those experienced in dissociative identity disorder (Ross 1997) or pathological gambling (Diskin and Hodgins 1999), but is not necessary for general and subclinical dissociative experiences to occur within a gambling context.

Follow-up research utilising eye-tracking techniques may represent a fruitful way to investigate the potential link between perceptual processing and response inhibition performance offered here. It is hypothesised that gambling stimuli would be attended to above and beyond ‘go’ and ‘no-go’ cues when gamblers are highly aroused, and that reduced attentional processing of ‘no-go’ cues would be predictive of poorer response inhibition performance.

### Caveats

#### Slot Machine Simulator

Whilst attempts were made to make the slot machine simulation in the current experiment as realistic as possible, such as the use of realistic stimuli and the use of real money with which to gamble, there were several structural and situational omissions when compared to slot machine gambling in a real-world setting. First, the slot machine here was simple in design, using a three-reel and single pay-line approach. The sophistication of slot machines is ever increasing, and it is not atypical to find slot machines in gambling venues in remote and online platforms to comprise five-, and even seven-reel designs. In addition, the number of pay-lines on slot machines can go beyond 2530 pay-lines, increasing the betting intensity of gamblers and providing more opportunity to both win and lose in shorter period of time. Whilst the pay-out structure in the present study purportedly allowed participants to win up to £10 on any one spin, this amount is small relative to the jackpot potential that can reach tens of thousands of pounds in real-world gambling settings. Finally, there are several in-game features lacking from the slot machine simulation used here, most notably the use of ‘nudge’ features and bonus rounds. All of these features likely impact the experience of gambling and effect the psychological processes relevant to gambling. However, it is likely, that these features are not conducive to self-control and would only further compound illusions of control and give rise to impulsive behaviours. For example, Parke et al. (2015) found that the opportunity to gamble and win larger amounts of money resulted in more impulsive choices and higher tolerance for uncertainty in a reflection impulsivity task.

One of the drawbacks of the controlled experimental design was the fact that participants were required to complete all trials in all conditions. In line with ethical guidelines (British Psychological Society 2018), participants were informed they could leave the overall study whenever they wanted and without reason, although they were asked to complete each gambling condition until the end, meaning that decisions to cease gambling was not a free choice per se. As a result, participants may have gambled for more or less time than they typically would in a real-world gambling setting, with likely implications for self-control. For example, if gamblers are restricted to a short period of time for their gambling session, then increased risk-taking and impulsive response styles may be a result of the temporal limitations imposed on their gambling. Conversely, if the experimental gambling session duration here was longer than typically experienced by gamblers, then fatigue effects may affect an individual’s ability to exercise self-control and sustain concentration and self-awareness. The duration of the gambling conditions was approximately 3.5 min for the fast speed condition, 5.5 min for moderate speed conditions, and 7.5 min for the slow speed conditions. According to Salis et al. (2015), a typical gambling session duration is approximately 8 min for machine gamblers. A typical amount of total time gambling in the present study was approximately 30 min long, although this was accompanied by a break of 5-min between each condition. Requiring a gambler to gamble longer than they normally would, could result in fatigue or boredom effects that have negative implications for self-control, although this limitation was likely offset by the forced breaks between conditions and the counterbalanced nature of trials.

Another limitation was the lack of control over gambling session duration across gambling conditions. This was a direct result of the event frequency manipulations whilst simultaneously controlling for the number of trials in each condition. As a result, it is hard to determine if factors predictive of response inhibition performance, such as levels of dissociative experience, are a product of the speed of play or by the time spent gambling.

### Subjective Arousal as a Proxy for Physiological Arousal

One of the assumptions made in the theoretical discussion presented here is that self-report measures of arousal were a proxy measure for physiological arousal. Cross-study comparisons typically demonstrate only a small to moderate level of convergence between self-report and physiological emotional responses (Mauss and Robinson 2009), although higher levels of convergence are typically found in research utilising within-participant designs (e.g., Mauss et al. 2005). It has also previously been argued that there is a temporal factor that effects the convergent validity of self-report with physiological measures. The fact that the SAM approach used here immediately follows the activity of interest likely adds to its convergent validity with other methodologies for assessing arousal. It has also been argued that the complex construct of emotional arousal cannot be captured by any one measure alone, and that it is multiply determined rather than characterised by a one-dimensional approach (Lang 1988). The explanation and discussion of results, whilst grounded in theoretical evidence, is therefore naturally subjected to a degree of interpretation. Replication would be a first priority, although further research would benefit from multiple concurrent measures of arousal within the gambling simulation, directly measuring physiological arousal using (for example) heart rate variability and galvanic skin responses, as well as variations in the self-report approaches to test for their convergence.

### Implications

The present study provides evidence that as the event frequency in electronic slot machine gambling increases, a gambler’s ability to exercise executive control is impaired. This was evidenced by a reduced capacity to withhold motor responses in an online test of response inhibition as speed of play increased during slot machine gambling. Arousal was a strong and negative predictor of response inhibition at fast speeds of play, although the effect of arousal on response inhibition appeared to be independent of a motor priming effect, which may suggest arousal impairs motor inhibition by a maladaptive biasing of a gambler’s perceptual processing in favour of gambling-related stimuli at the expense of environmental cues necessary for exercising self-control. Evidence suggests there is an interaction effect between the speed of slot machine gambling and the psychological factors that predict response inhibition performance. As speed of play is slowed, the relative predictive strength of arousal in inhibition is reduced, and levels of dissociative experience become the dominant and negative predictive of response inhibition. However, this speed-induced dissociation account must be treated with caution because the experimental design meant it was not possible to separate the effects of speed of play and duration of play in the slow speed condition. This means that increased dissociation may have resulted from a longer period of slot machine play at slow speeds, rather than as a direct result of the decreased speed of play. Nevertheless, both the arousal route and dissociation route to reduced motor inhibition are consistent with the notion that inefficient perceptual processing may represent an underlying mechanism that results in impaired response inhibition during gambling. At fast game speeds, if elevated arousal results in enhanced processing of gambling-related stimuli, then this should leave fewer resources available for effortful self-control. Similarly, at slower game speeds, although associated with improved inhibition performance compared to fast speeds, if gamblers are experiencing greater levels of dissociation, environmental cues designed to aid self-control are less likely to be processed.

If this is the case, harm-minimisation approaches during gambling should aim at adjusting the salience of cues that may assist self-control. Whilst ‘no-go’ cues are a feature specific to this gambling simulation, other cues exist in gambling environments that may help a gambler exercise greater levels of self-control. Such approaches may include making clocks and monetary spend displays more salient to ensure they are regularly processed and attended to by gamblers, with the intention of making them more self-aware of the amount of time and money they have spent gambling. In terms of motor impulsivity, the intermittent implementation of stop cues within slot machines may themselves offer a way of enhancing response inhibition. Although response inhibition was impaired as game speeds increased, an impaired perceptual processing when aroused account predicts that if the salience of stop cues were enhanced, then this should offset some of the negative effects of elevated arousal on response inhibition during gambling.

Without consideration for wider contextual issues, an obvious solution might be calls for legislative action to reduce the maximum speed of electronic slot machines, as slower speeds have been shown to be less detrimental to self-control with the present study. However, reducing the speed of play comes at the price of reducing gambling enjoyment, as evidenced here. As a result, in more liberal societies such as the UK, such policies are less likely to be publicly accepted and may potentially be viewed by gamblers as an overly paternalistic approach to harm reduction. In addition, there is potential for perverse and unintended consequences for gambling behaviour resulting from a cap on gambling machine speed. If speed is reduced, this could result in compensatory gambling behaviours, where gamblers play more gambling lines, bet larger amounts, and play for longer periods of time on slot machines to compensate for the reduced speed of play. Therefore, it is important that further academic research into these potential consequences is the prerequisite to any wide-scale changes in gambling policy.

## Conclusion

Motor response inhibition represents a single, and yet important aspect of self-control within a gambling context. Impulsivity by definition is the execution of action without foresight or planning and therefore represents an undesirable response style within a gambling context where there is potential for gamblers to experience gambling-related harm. The more that gambling decisions are made through rational and conscious choice, the more likely it is that gambling will remain an enjoyable and safe leisure pursuit. Conversely, the more frequent that actions are performed based on impulsive execution, the more likely that this will ultimately lead to behavioural markers of harm, including excessive time and money spent gambling and reduced ability to quit the game at appropriate times. Problematic for the gambling industry is that the present study found that increased speed of play during slot machine gambling results in impairments in self-control during gambling among a sample of non-problem gamblers. This demonstrates that structural characteristics of gaming machines, in this case speed of play, can produce impulsive behaviours independent of predispositional vulnerability amongst gamblers.
